# Syncytiotrophoblast extracellular vesicles – Circulating biopsies reflecting placental health

**DOI:** 10.1016/j.placenta.2016.11.008

**Published:** 2017-04

**Authors:** Dionne Tannetta, Gavin Collett, Manu Vatish, Chris Redman, Ian Sargent

**Affiliations:** a Department of Food and Nutritional Sciences, University of Reading, PO Box 226, Whiteknights, Reading RG6 6AP, UK; b Nuffield Department of Obstetrics and Gynaecology, University of Oxford, Level 3, Women's Centre, John Radcliffe Hospital, Oxford OX3 9DU, UK

**Keywords:** Syncytiotrophoblast extracellular vesicles, Preeclampsia, Microvesicle, ExosomeSyncytiotrophoblast biopsy

## Abstract

The ability to directly monitor the status of the placenta throughout pregnancy would be a major advance in both general and personalized obstetric care, allowing treatments to be tailored to the dynamic changes that can occur in gestation. Syncytiotrophoblast extracellular vesicles (STBEV) are membrane bound vesicles, released from the surface of the placenta directly into the maternal circulation, in the form of exosomes, microvesicles and apoptotic bodies. They carry many syncytiotrophoblast derived factors such as proteins, lipids, glycans and nucleic acids, which together could dynamically signal to the mother the status of the placenta. We review STBEV research and discuss the potential for STBEV to be used as circulating syncytiotrophoblast biopsies, accessible via a simple blood sample throughout pregnancy, giving a real-time readout of syncytiotrophoblast health. We also highlight advances in the use of extracellular vesicles as circulating tumour derived biopsies in the field of cancer research, which could prove beneficial to obstetric care.

## Highlights

• Syncytiotrophoblast release extracellular vesicles (STBEV) directly into the maternal circulation, during normal pregnancy and in increased amounts in preeclampsia, in the form of exosomes, microvesicles and apoptotic bodies.
• STBEV carry many syncytiotrophoblast derived factors such as proteins, lipids, glycans and nucleic acids, the composition of which can change with syncytiotrophoblast stress.
• Circulating STBEV are therefore placental “biopsies” accessible throughout gestation, giving a real-time readout of syncytiotrophoblast health.
• STBEV have great potential as biomarkers for monitoring heterogeneous syndromes such as PE and other forms of placental compromise/stress.

## 1 Introduction

The syncytiotrophoblast (STB) is fundamental to the establishment and maintenance of a healthy pregnancy. Therefore, impaired functional capacity of the STB impacts directly on fetal development and maternal adaptations to pregnancy. An extreme example is preeclampsia (PE) where the only effective treatment is removal of the placenta [1]. Acute or chronic changes that occur through physiological or pathological mechanisms can lead to impaired STB function. Such changes will probably vary within one placenta and between individual pregnancies, although certain pathological processes may be common to specific complications [2]. A real time read out of STB functional status, accessible throughout gestation, would be invaluable in monitoring STB performance and permit direct measurement of placental health.

Extracellular vesicles (EV) are membrane bound, cell derived particles released by many different cell types. The term EV encompasses exosomes (∼100 nm in diameter), microvesicles (0.1–1 μm) and apoptotic bodies (0.5–5 μm), released as part of both normal physiological functions, such as haemostatic regulation and cell maintenance, and as a consequence of pathologies, such as cancer, cardiovascular disease and inflammatory disorders [3], [4], [5]. Cellular responses such as activation, stress and death alter the molecular fingerprint of the EV cargo, making them potential biopsies of inaccessible tissues and allowing measurement of multiple biomarkers derived from a specific source [6]. STB releases EV (STBEV), containing a complex cargo of RNAs, proteins, glycans and lipids, from the apical surface directly into the maternal circulation in normal pregnancy and PE, fuelling interest in their use as biomarkers of placental wellbeing [7], [8], [9], [10], [11], [12].

## 2 Types of STBEV and their role in normal pregnancy and PE

The STB layer releases exosomes, microvesicles and apoptotic bodies that pass through the maternal pulmonary capillary bed and out into the systemic circulation [7]. In normal pregnancy, some, if not all of the STBEV subtypes are released constitutively and are thought to have a role in inducing maternal adaptive changes, which include suppressed maternal cell mediated immunity [13]. Possible immunoregulatory factors carried by STBEV include apoptosis inducers FasL and TRAIL, the NKG2D receptor modulators MIC A/B and ULBPs and the T cell regulators B7-H1, B7-H3 and HLA-G5 [14], [15], [16]. Interestingly, changes in STBEV immunoregulatory activity have also been seen over the course of normal pregnancy, with first trimester STBEV being more pro-inflammatory than those derived from term placenta [17]. Such observations highlight the potential physiological changes in STBEV phenotype and cargo over the course of gestation although, since first trimester placental tissue is obtained after pregnancy termination, artefactual stresses could have a significant impact on the observed changes in STBEV biological activity.

The placenta plays a key role in triggering systemic inflammation, endothelial dysfunction and hypercoagulation that underlie the maternal symptoms of PE. The maladaptations underpinning severe disease occur early in pregnancy, with inadequate remodelling of the uteroplacental spiral arteries leading to compromised placental perfusion [18], [19]. Impaired placental function may ensue and, in severe cases, damage due to inflammatory, oxidative and ER stresses occurs, all of which could stimulate the release of excess STBEV with a pathological phenotype and cargo [20], [21]. This scenario is supported by several studies, including our own work showing that treatment of BeWo choriocarcinoma cells with tunicamycin, to induce ER stress, stimulates the increased release of EV, larger in size and carrying an increased cargo of danger associated molecular pattern (DAMP) molecules (G Collett unpublished observation), also known as alarmins, which are key players in triggering a sterile inflammatory response [22]. Using STBEV prepared from *ex vivo* placental lobe dual perfusion and cultured term placental explants, STBEV released by PE placentas (PE-STBEV) were also found to be significantly larger than those from normal placentas [17], [21]. *In vitro*, PE-STBEV differentially affect immune cell, endothelial cell and platelet activation, suggesting a role in the maternal systemic inflammatory response, endothelial dysfunction and hypercoagulation, which are part of the maternal syndrome of PE [17], [23], [24], [25]. In summary, the spectrum of STBEV subtypes released in PE, changes from smaller exosomes towards larger microvesicles and apoptotic bodies, which carry an altered repertoire of biologically active factors, consistent with increased placental stress in PE. However, the biological effects of STBEV isolated from placentas of normal, early onset PE and late onset PE pregnancies have not been systematically compared, which would add much to our understanding of the underlying placental pathology.

## 3 Circulating STBEV in normal pregnancy and PE

Circulating levels of EV reflect the balance between the rates of release and clearance. In animal models EV are rapidly cleared from the circulation, predominantly by liver and spleen, suggesting that a significant increase in EV release (or decrease in clearance rate) would be required to impact circulating levels [26], [27]. STBEV are taken up by circulating monocytes, B cells and platelets [25], [28], but no data are available on clearance of STBEV by organs (e.g. lungs, heart, kidneys, liver and spleen). It is also not known whether STBEV leave the circulation via interstitial fluid into the lymphatic system or to what extent circulating STBEV represent the population first released by the placenta. This highlights the need for comparisons between STBEV originally released by the STB (e.g. *ex vivo* derived STBEV from placental dual perfusion or those present in uterine vein blood samples, which would most closely reflect the total STBEV population) and peripheral circulating STBEV.

**Fig. 1:**
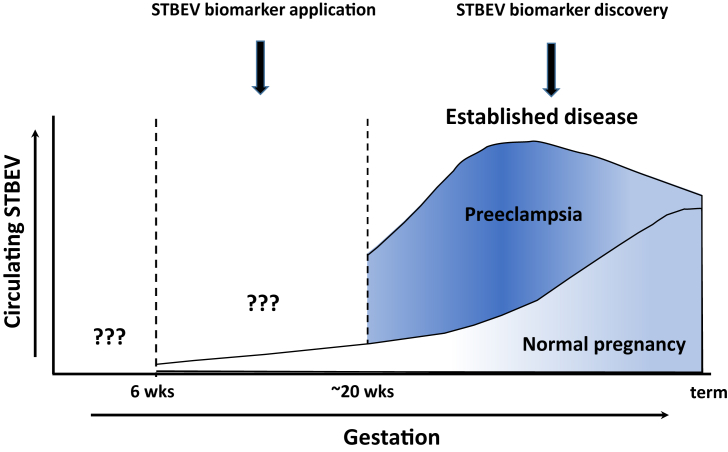
Schematic diagram summarising current literature on circulating levels of syncytiotrophoblast derived extracellular vesicles (STBEV) in normal pregnancy and preeclampsia. The intensity of the shading represents the incidence of pathological changes in the placenta and when corresponding changes in STBEV composition may be most evident.

In normal pregnancy, circulating STBEV are detectable in the first trimester by both placental alkaline phosphatase (PLAP; STB marker) immunoassay and flow cytometry (both free and bound to monocytes), with levels progressively rising over the course of gestation and peaking at term [29]. In established PE, circulating STBEV levels are significantly higher in early onset PE (<34 weeks) compared to matched normal pregnancy [29], [30]. However, it is not known if increased STBEV levels also predate clinical symptoms of PE (Fig. 1). Analysis of circulating STBEV subtypes in normal pregnancy and PE is also not available owing to a lack of reliable subtype specific markers. Circulating levels of EV that are double positive for PLAP and CD63 (marker enriched in exosomes) were reported to be detectable at 6 weeks and to rise over the subsequent course of normal gestation, suggesting that STB derived exosomes can reach the systemic circulation prior to the establishment of blood flow into the intervillous space (∼10 weeks gestation), although the mechanism enabling this transfer is not known [31], [32].

Quantification of circulating STBEV number is dependent on the sensitivity of the method used. In theory immunoassays using PLAP for capture and/or substrate cleavage should give a measure of the total PLAP positive STBEV population. This assumes that any differences in comparisons are due to changes in STBEV number rather than PLAP expression. Using nanoparticle tracking analysis (NTA) and fluorescence-NTA; techniques able to track individual EV in the size range of 50 nm to 1 μm, we found lower PLAP positivity in placental perfusate derived exosomes compared to preparations enriched for microvesicles, suggesting lower PLAP expression on STB derived exosomes [33]. This may be due to the smaller surface area of exosomes compared to microvesicles, or selective packaging of PLAP into microvesicles. We have also found PLAP expression to be significantly lower for PE-STBEV, compared to those derived from healthy pregnancy placentas, using mass spectrometry (measures total (surface and intravesicular) PLAP; D Tannetta unpublished observation), Western blotting (total PLAP) and flow cytometry (surface PLAP), suggesting that PLAP positivity may give an underestimate of STBEV number in PE [21]. By flow cytometry, which has an EV detection limit of 300–500 nm (favouring detection of microvesicles and apoptotic bodies), PLAP positive STBEV have been estimated to comprise ∼0.5–5% of the total EV population [34], [35]. Finally, the total number of plasma exosomes, isolated using density gradient ultracentrifugation and quantified by NTA, has been estimated to increase 50 fold in the first trimester of pregnancy, although the contribution of placenta derived exosomes to this increase is currently not known [31]. Therefore, although there is strong evidence that STBEV can be found in the maternal circulation from 6 weeks onwards, quantification of their absolute number, given these findings, remains challenging.

## 4 STBEV as STB biopsies

STBEV have promising attributes for being effective STB biopsies. They are released directly into the maternal circulation, so minimal invasive sampling is required. In theory, circulating STBEV are derived from the entire surface of the placenta; therefore, unlike a traditional biopsy which involves small tissue sampling areas, circulating STBEV would give a much better overall representation of STB status. STBEV also express STB specific surface markers such as PLAP that enable their exclusive isolation [21]. STBEV carry a varied cargo derived directly from the STB, but their intravesicular cargo is also protected by a phospholipid bilayer that could help to improve detection of low abundance biomarkers and those with a short half-life, such as mRNAs [36]. Given their rapid clearance it is likely that they represent an up-to-date picture of current STB health [26], [27], [37]. Also, longitudinal studies measuring circulating levels of STB derived exosomes, microvesicles and apoptotic bodies, would not only reveal the dynamics of STBEV subtype release but give a non-invasive analysis of STB turnover, and how it changes under stress conditions. Finally, if STBEV release and cargo can reflect pathological changes, STBEV have great potential as biomarkers for personalized diagnostics, allowing the tailoring of treatments to a particular patient's circumstances. This could prove to be important for the effective treatment of heterogeneous syndromes such as PE.

A major limitation in STBEV research is the paucity of reliable markers to discriminate STBEV subtypes. So it is not known whether a specific STBEV subtype or the total STBEV population would be a better circulating STB biopsy. The study of *ex vivo* placenta derived preparations of STBEV has important limitations, especially with early onset PE requiring preterm delivery. Normal preterm controls are not readily available so differences may reflect gestational age rather than pathology. By necessity, such investigations use placental material collected when the disease is established, but predictive biomarkers should be present prior to clinical symptoms (Fig. 1). The study of blood derived STBEV would allow both retrospective matching of normal pregnancy and PE samples and analysis of STBEV biomarker potential prior to onset of disease. However, current variation in the methods used to collect samples and isolate and characterise STBEV could all impact on STBEV composition. Without standard procedures, this makes the validation of results across laboratories much harder.

## 5 Isolation of circulating STBEV

Ideally, an effective method for the isolation of circulating STBEV would require routine blood handling techniques, minimal sample preparation and would give a high yield of undamaged pure STBEV from a small sample volume. In reality, the targeted isolation of phenotypical EV from a blood sample requires specialised isolation and detection methods, due to the subcellular nature of EV and the complexity of blood which, apart from cells, also contains EV from multiple sources (including platelets, red blood cells, leucocytes and endothelial cells) as well as numerous soluble factors with the potential to interfere in isolation methods and downstream analyses (such as STBEV cargo determination using transcriptomic and proteomic approaches). However, prior to isolation, venepuncture and blood handling, type of anticoagulant and fasting status are just some of the pre analytical variables that can affect the EV composition of a sample [38]. Multiple processing steps can also lead to accumulating losses of EV. Therefore, to minimalize the impact of such losses, the level of purity required for any subsequent analyses should be taken into account when choosing an appropriate isolation technique [6], [39].

Crude preparations of total EV can be quickly obtained by precipitation techniques followed by low speed centrifugation [40]. More routinely used methods to isolate EV are based on size or density, such as differential centrifugation, density gradient ultracentrifugation, microfiltration and size exclusion chromatography [40], [41]. For even greater purity and enrichment of particular EV phenotypes, immunocapture using antibodies to specific surface antigens has also proved effective [42]. There are advantages and disadvantages to each method that have been comprehensively reviewed [6], [42], [43], [44]. Briefly, centrifugation techniques have been widely used to isolate EV from various sample types and have also been used to fractionate EV into microvesicles and exosomes, such as STBEV from placental perfusate and dendritic cell EV [33], [45]. However, EV isolated using techniques such as precipitation and ultracentrifugation can be contaminated with protein aggregates and, in the case of blood derived samples such as plasma, high density lipoproteins, which can interfere in downstream analyses [43], [46]. Size exclusion chromatography (SEC) is becoming the preferred method for EV isolation because it is a quick and gentle method that gives high yields of EV with good separation from soluble proteins [41]. However, for rapid diagnostic use, highly enriched EV of a particular phenotype can be obtained using microfluidic devices and immunocapture onto beads, magnetic nanoparticles or chips by targeting those EV expressing particular surface markers [42].

PLAP has proved to be a very useful marker for STB derived EV detection, however during pregnancy soluble PLAP circulates at high levels and could compete with STBEV bound PLAP for immunocapture. Also, as described above, STBEV associated PLAP is significantly reduced in PE [21]. Therefore, although PLAP is a robust STBEV marker, its use to determine STBEV number may lead to an underestimation in PE. Partially enriched preparations of STBEV could also be obtained by using a marker expressed on STBEV and EV of other cell types affected in PE such as endoglin; expressed on STBEV in normal pregnancy and PE and also carried by endothelial cell derived EV, the circulating levels of which are increased in PE [21], [47], [48]. As the STB is the largest epithelium in direct contact with maternal blood during pregnancy, an alternative strategy could be to isolate epithelial derived EV. The oncoprotein mucin-1 is a transmembrane glycoprotein highly expressed on the apical surface of polarised epithelia, including STB where expression of mucin-1 is significantly increased in PE [49]. Mucin-1 is also expressed on the surface of EV released by several tumour types both *in vivo* and *in vitro* [50], [51], [52]. We have found that STBEV contain mucin-1 at significantly increased levels in PE, showing promise as a circulating STBEV marker in PE (D Tannetta, R Dragovic & G Collett unpublished observation).

## 6 Parallels with cancer EV

Cancer cells also release EV that are implicated in tumour progression and metastasis such as inflammation, modulation of cell mediated immunity and angiogenesis [53], [54]. Here, biomarkers in personalized care cover multiple stages in the development and treatment of cancer including screening, early diagnosis, prediction, prognosis and monitoring disease progression [54]. Indeed, cancer displays all the problems of heterogeneity both between and within patients and the need for a personalized approach to treatment, which are likely operative in PE.

The cancer field leads the way in the clinical application of EV analysis, in particular of exosomes, and in the use of exosome derived mRNA, miRNA and protein based assays [44], [54]. Traditional methods of isolating exosomes, such as differential centrifugation, are not suitable for routine clinical application owing to low sample throughput and low exosome yield and purity. The need for highly sensitive and specific detection systems due to the relatively low abundance of tumour specific exosomes in a heterogeneous mix of circulating EV also poses a challenge, analogous with the detection of circulating STBEV during early pregnancy.

Novel techniques for the capture and analysis of cancer derived EV have and continue to be developed. This includes methods such as on-chip immunoelectrophoresis, which identifies changes in the zeta potential of individual cancer EV following binding of EV specific antibodies [55]. Nanoplasmonic technology has been utilised to capture ovarian cancer exosomes using epithelial marker specific antibodies [56]. An acoustic nanofilter system has also been developed that separates EV according to size and density using differential acoustic force generated by ultrasound waves [57]. Finally, serum colorectal cancer derived EV have been detected directly in patient blood samples using Exoscreen, a technique whereby cancer specific EV are captured using two types of antibodies and detected by photosensitizer beads [58]. Interestingly, identification of novel disease biomarkers may not be necessary as exosome association may also improve existing biomarker specificity. For example, exosome associated levels of the validated prostate cancer biomarker prostate-specific antigen (PSA), have been found to have higher disease specificity than total circulating and urinary levels [59], [60]. Likewise, given the controversy over the variability in reported levels and cellular source of circulating sFlt-1 in PE, STBEV associated sFlt-1 could be a more informative measure of placenta derived sFlt-1 [61]. Such innovations may aid the pregnancy field to overcome limitations such as low numbers of circulating STBEV during early pregnancy and specificity of some circulating PE markers, enabling better utilisation of STBEV for clinical applications.

## 7 Conclusions

There is increasing evidence that STBEV have huge potential as circulating STB biopsies, easily accessible throughout gestation and as a real time reflection of STB status. Their complex cargo of mRNA, miRNA, proteins, glycans and lipids gives a potentially rich source of biomarkers in complications involving placental dysfunction. Most focus has been on STBEV as biomarkers to predict or diagnose PE, with the cargo giving a PE ‘finger print’ reflecting specific pathological mechanisms such as inflammatory, oxidative and ER stresses. However, there is now a pressing need to relate data on STBEV and their cargo, generated *ex vivo* from normal and PE affected placentas to STBEV found in blood samples. Moreover, a thorough profiling of circulating STBEV during the course of normal pregnancy is also needed to facilitate the identification of pathological changes. This would be greatly benefitted by the development of an efficient, reliable and clinically feasible extraction method. Finally, STBEV show promise in the clinical monitoring of PE, but STBEV analysis may also be applicable to other forms of placental compromise/stress and even epigenetic changes that may alter both short and long term fetal outcomes.

## Conflict of interests

The authors declare no conflict of interest.
